# Overcoming establishment thresholds for peat mosses in human‐made bog pools

**DOI:** 10.1002/eap.2359

**Published:** 2021-05-30

**Authors:** Ralph J. M. Temmink, Peter M. J. M. Cruijsen, Alfons J. P. Smolders, Tjeerd J. Bouma, Gregory S. Fivash, Wouter Lengkeek, Karin Didderen, Leon P. M. Lamers, Tjisse van der Heide

**Affiliations:** ^1^ Aquatic Ecology and Environmental Biology Institute for Water and Wetland Research Radboud University Heyendaalseweg 135 Nijmegen 6525 AJ the Netherlands; ^2^ Department Coastal Systems Royal Netherlands Institute of Sea Research and Utrecht University Landsdiep 4 't Hortje (Texel) 1797 SZ the Netherlands; ^3^ B‐WARE Research Centre Toernooiveld 1 Nijmegen 6525 ED the Netherlands; ^4^ Department of Estuarine and Delta Systems Royal Netherlands Institute of Sea Research and Utrecht University Korringaweg 7 Yerseke 4401 NT the Netherlands; ^5^ Delta Academy Applied Research Centre HZ University of Applied Sciences Vlissingen the Netherlands; ^6^ Faculty of Geosciences Department of Physical Geography Utrecht University Princetonlaan 8a Utrecht 3584 CB the Netherlands; ^7^ Conservation Ecology Group Groningen Institute for Evolutionary Life Sciences University of Groningen Nijenborgh 7 Groningen 9747 AG the Netherlands; ^8^ Bureau Waardenburg Varkensmarkt 9 Culemborg 4101 CK the Netherlands

**Keywords:** alternative stable states, rewetting, raised bog, peat moss, *Sphagnum*, terrestrialization

## Abstract

Globally, peatlands have been affected by drainage and peat extraction, with adverse effects on their functioning and services. To restore peat‐forming vegetation, drained bogs are being rewetted on a large scale. Although this practice results in higher groundwater levels, unfortunately it often creates deep lakes in parts where peat was extracted to greater depths than the surroundings. Revegetation of these deeper waters by peat mosses appears to be challenging due to strong abiotic feedbacks that keep these systems in an undesired bare state. In this study, we theoretically explore if a floating peat mat and an open human‐made bog lake can be considered two alternative stable states using a simple model, and experimentally test in the field whether stable states are present, and whether a state shift can be accomplished using floating biodegradable structures that mimic buoyant peat. We transplanted two peat moss species into these structures (pioneer sp. *Sphagnum cuspidatum* and later‐successional sp. *S. palustre*) with and without additional organic substrate. Our model suggests that these open human‐made bog lakes and floating peat mats can indeed be regarded as alternative stable states. Natural recovery by spontaneous peat moss growth, i.e., a state shift from open water to floating mats, is only possible when the water table is sufficiently shallow to avoid light limitation (<0.29 m at our site). Our experiment revealed that alternative stable states are present and that the floating structures facilitated the growth of pioneer *S. cuspidatum* and vascular plants. Organic substrate addition particularly facilitated vascular plant growth, which correlated to higher moss height. The structures remained too wet for the late‐successional species *S. palustre*. We conclude that open water and floating peat mats in human‐made bog lakes can be considered two alternative stable states, and that temporary floating establishment structures can induce a state shift from the open water state to peat‐forming vegetation state. These findings imply that for successful restoration, there is a clear water depth threshold to enable peat moss growth and there is no need for addition of large amounts of donor‐peat substrate. Correct species selection for restoration is crucial for success.

## Introduction

Peatlands provide vital ecological and socioeconomic services at a global scale (Joosten and Clarke 2002). While they only account for ~3% of the terrestrial surface (Yu et al. 2010), they store ~20% of the global soil organic carbon (Scharlemann et al. 2014, Nichols and Peteet 2019, Günther et al. 2020). Furthermore, peatlands retain freshwater, provide clean drinking water and biodiversity (Limpens et al. 2008, Lamers et al. 2015). However, peatlands are being drained at a large scale to facilitate agriculture, forestry, and peat extraction (Swindles et al. 2019). Drainage leads to the emission of greenhouse gases, deterioration of groundwater and surface water quality, and subsidence of peat soils (Schothorst 1977, Verhoeven and Setter 2010, Lamers et al. 2015). However, extraction and drainage also creates unnatural landscapes with altered hydrology and variable thickness of the remaining peat layer (Haapalehto et al. 2014).

**Fig. 1:**
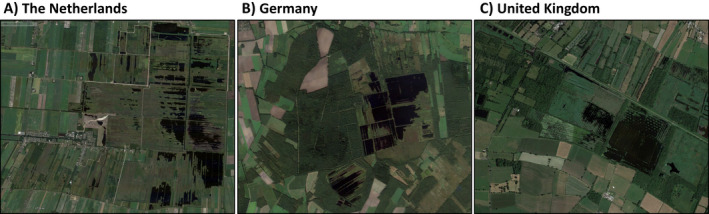
Examples of unnatural open water in former natural raised bogs. (A) Bargerveen in the Netherlands, (B) Tister Bauernmoor in Germany, and (C) Shapwick Heath in the United Kingdom. Maps from Google Earth.

To restore peat‐forming vegetation in degraded peat bogs (formerly dominated by peat mosses, *Sphagnum* spp.), the construction of dams that reduce water loss by lateral flow and maintain a permanently high groundwater table is an often applied approach (Schumann and Joosten 2008, Parry et al. 2014, Altenburg et al. 2017). Although this successfully elevates groundwater tables, it typically also causes deeply excavated areas to turn into relatively deep bog lakes. These bog lakes are frequently without vegetation even decades after rewetting. Numerous examples of remnant bogs with large bodies of open water can be found in the Netherlands, Germany, the Baltic States, the United Kingdom (Fig. 1), and in North America (Quinty and Rochefort 2000). This implies that although the restoration measures are partly successful, large parts of the previously drained areas remain as persistent open water without the targeted bog vegetation.

In general, persistent degraded states often occur in disturbed ecosystems that are naturally controlled by positive feedback mechanisms. Such feedbacks are typically generated by spatially dominant habitat‐forming organisms, which modify their surroundings to their own benefit by reducing physical stress or increasing resource availability (Stachowicz 2001). However, these beneficial modifications often only work beyond a certain minimum density or patch‐size of the habitat modifier, yielding a positive (i.e., self‐reinforcing) feedback on its own growth. Unsuccessful colonization (e.g., too low density or patch‐size) of the species may cause alternative stable states (Scheffer et al. 2001, van der Heide et al. 2007). This implies that, either a state that is dominated by the habitat‐modifying species or an alternative (often bare) state is stable under the same environmental conditions. Importantly, natural recovery in such situations is difficult once the abundance of the habitat‐modifying species drops below a critical density or patch‐size threshold. Well‐known examples of ecosystems with habitat‐modifying species generating strong positive feedbacks are submerged aquatic vegetation in shallow lakes and coastal seagrasses that reduce turbidity through trapping of suspended particles and stimulate phytoplankton grazing by providing refuge to zooplankton (Scheffer et al. 2001, van der Heide et al. 2007, 2010).

In bogs, peat mosses (*Sphagnum* spp.) are the dominant habitat‐modifying species (Van Breemen 1995). As they increase in biomass, density, and patch‐size, they increasingly alter their environment by retaining nutrient‐poor rainwater, acidification, and the accumulation of organic material (Van Breemen 1995, Lamers et al. 2000, Soudzilovskaia et al. 2010). In this way, mosses improve their own growing conditions with increasing moss abundance, yielding a positive feedback. Peat mosses colonize bog lakes through a process called terrestrialization. This process naturally occurs via the formation of a stable, permanent floating mat consisting of live mosses, structuring vascular plants, and dead organic material, which can initiate at the lake bottom, from the shore or from free‐floating mosses. The mosses produce oxygen from photosynthesis, while degradation of organic matter by its associated microbial community produces methane (CH_4_) and carbon dioxide (CO_2_). Buoyance is generated by oxygen that accumulates inside the mosses and the other gasses that are trapped inside and beneath the peat mat (Smolders et al. 2002, Tomassen et al. 2003*b*, 2004; Appendix S1: Fig. S1). Once afloat, the peat mosses are exposed to atmospheric CO_2_ and have ample light for growth; conditions that further stimulate their growth and subsequent organic matter accumulation.

In deep (human‐made) bog lakes, vegetation is often persistently lacking, as peat moss colonization from the bottom is hampered by light limitation due to humic substances in the water column (Smolders et al. 2003). Additionally, colonization by free‐floating mosses is also hampered, as these mats typically do not become thick enough during the growing season to sustain buoyancy in winter when production of oxygen, CO_2_ and CH_4_ are all decreased (Smolders et al. 2003, Tomassen et al. 2004). Once the mosses are at the bottom, light and dissolved CO_2_ levels can be too low to allow sufficient photosynthesis and oxygen production required for resurfacing in the next growing season (Paffen and Roelofs 1991, Smolders et al. 2003). Often, the threshold of 5% light, the minimum level required for submerged peat moss growth, occurs at a depth 0.2–0.5 m when waters are highly colored (Streefkerk and Casparie 1989, Money and Wheeler 1999, Smolders et al. 2003). This implies that mat formation beyond this depth is not possible. In addition, peat moss growth is also limited by low dissolved carbon availability (<750 µmol CO_2_/L; Paffen and Roelofs 1991, Patberg et al. 2013). Apart from issues with light and carbon limitation, early forming peat mats that manage to become afloat can also be hampered by wind‐generated waves that break the mats apart, a factor that may be particularly important in larger lakes where fetch lengths are long (Wheeler and Shaw 1995, Smolders et al. 2003). These difficulties for re‐establishment of the original peat system raises the question to what extent positive feedbacks, and the potential for alternative stable states, play a role in the persistence of these human‐made bog lakes.

The idea for the existence of alternative stable open water and floating mat states in deep bog lakes is not only supported by observations. In a number of small‐scale bog‐restoration research projects, aiming to revegetate deep bog lakes, this was achieved this by introducing poorly humified peat mats with a living top layer (white peat, German: *Bunkerde*). In these cases, the mats became buoyant due to CO_2_ and CH_4_ formation, thereby overcoming apparent CO_2_‐ and transparency‐related establishment thresholds for peat mosses (Money and Wheeler 1999, Smolders et al. 2002, Tomassen et al. 2003*b*, 2004). However, this approach has clear downsides, because (1) pristine donor sites are damaged by the harvest of transplant material and (2) the quality of white peat can differ greatly; from slow degrading nutrient‐poor pristine bog peat dominated by peat mosses to dry and eutrophic top soils dominated by *Molinia* that rapidly degrade (Money and Wheeler 1999, Lamers et al. 2000). Consequently, restoration measures to aid the terrestrialization of deep human‐made bog pools, without using unsustainable black or white peat, are currently lacking.

In this study, we therefore (1) explore whether open water and floating peat mats can indeed be considered two alternative stable states, and (2) test if establishment thresholds caused by low transparency, stress from CO_2_ limitation and wind‐generated waves can be overcome by using temporary floating support structures. First, to test whether the hypothesized alternative stable states are theoretically possible, we constructed a minimal model (i.e., a simplified model for exploring processes and related system dynamics in isolation) based upon a combination of literature data (Boatman 1977, Hayward and Clymo 1983, Money 1995, Smolders et al. 2002, 2003, Rochefort et al. 2003) and field measurements in Fochteloërveen, the Netherlands. Specifically, the model focuses on light limitation as the simplest possible explanation for alternative stable states, thus ignoring other potentially exacerbating factors such as CO_2_ limitation at the bottom or waves once the mat is afloat. Hence, it describes the relation between peat moss growth and white peat accumulation, dependent on light availability, which in turn depends on water depth, water transparency and on whether the peat mat is buoyant or not. We hypothesize that the model, with parameters calibrated using empirical data, can generate alternative stable states in a range of realistic water transparencies and depths.

**Fig. 2:**
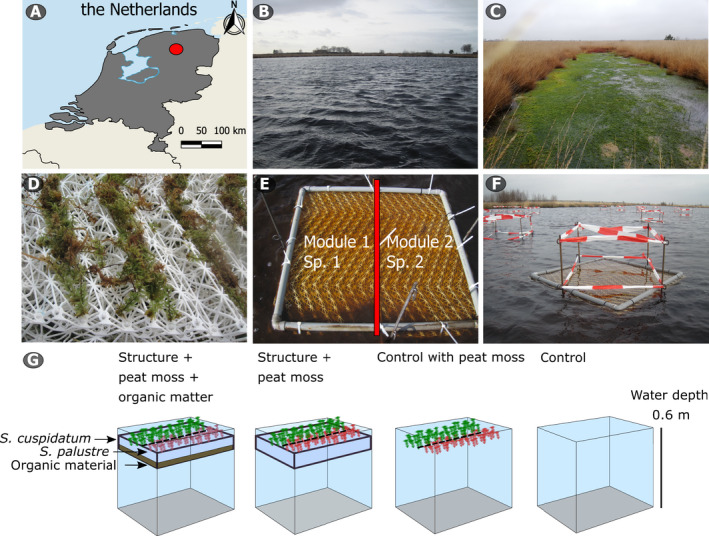
Field site and experimental units. (A) The Netherlands, where the Fochteloërveen is indicated by a red circle, (B) open water without terrestrialization, (C) floating peat mat consisting of *Sphagnum* *cuspidatum*, (D) peat moss application to the establishment structure, (E) setup of two modules containing different species, and (F) experimental plots after setup with caution tapes to exclude birds. (G) Overview of the four original experimental treatments, where peat moss species are indicated with different colors. The control with peat moss was omitted (see Materials and Methods: Field experiment for details). Pictures: R. J. M. Temmink. Map made with Natural Earth.

Next, to investigate whether alternative stable state conditions are present in the field and if they can be overcome, we tested a novel restoration framework (Temmink et al. 2020). In an experiment, we use biodegradable floating establishment structures (hereafter, structures for brevity) that mimic buoyant peat. In the structures, peat mosses have ample light and CO_2_ for growth, as well as structural reinforcement to increase resistance against small waves (Fig. 2D, E). We expect that the temporary structures allow mosses to proliferate and ultimately form dense floating mats. The structures can naturally degrade once the mats generate buoyancy themselves (Tomassen et al. 2004), are sufficiently coherent to resist waves, and can act as nuclei for lateral growth. To test the restoration potential of our framework, we selected a human‐made bog lake with highly colored (E_450_ = 0.17, 5% light penetration threshold at 0.3 m) and carbon limited water layer, which is too deep (~0.6 m) to support peat moss growth at the bottom. We selected the pioneer S*phagnum cuspidatum* and late‐succession species *S. palustre* as model species (Daniels and Eddy 1985, Frahm and Frey 2003) that were transplanted inside structures with and without organic substrate. We hypothesize that (1) the lake has two alternative state states as described by the model, (2) the structure physically supports peat mosses and ensures sufficient light and CO_2_ and enables to mosses to resist waves, and (3) when enriched with organic material it provides additional CO_2_ and nutrients, further stimulating growth and peat mat development.

## Materials and Methods

### Model description

Many lakes in rewetted raised bogs have either been fully colonized by vegetation or persist for decades without any significant vegetation development (Smolders et al. 2003, Patberg et al. 2013). We constructed a minimal model that explores the basic nature of the terrestrialization process in a qualitative manner (Couwenberg and Joosten 2005, van der Heide et al. 2007, 2010). Specifically, this model describes the relation between light, peat moss growth, and white peat accumulation. Peat moss growth, and consequently white peat accumulation, is dependent on light availability, which in turn depends on water depth, water transparency and on whether the peat mat is buoyant or not. Model parameter settings were derived from direct field measurements or literature data (Table 1, Appendix S1: Table S1). The change in the living peat moss layer over time is described as

(1) $$\frac{\mathrm{dS}}{\mathrm{dt}}=r\times \text{fl}\times S‐\left(\left(r‐m\right)/K\right)\times S^{2}‐m\times S.$$

**Table 1:** Variables and default parameter settings of the peat moss model.

	Default value	Unit	Description	Sources
Variables				
*S*		m	living peat moss layer thickness	
*M*		m	mat thickness	
Parameters				
*d*	0.001	per day	mat decay rate	based on empirically measured mat thickness of 0.5 m
*D*_max_	0.5	m	depth of the lake	estimated from field data
*I*_0_	500	mol·m^−2^·s^−1^	growing‐season‐averaged irradiance at the surface	Royal Dutch Meteorological Institute (2019)
*I_k_*	200	mol·m^−2^·s^−1^	half‐saturation irradiance constant	Titus and Wagner (1984)
*K*	0.1	m	carrying capacity of *S*	Clymo (1970)
*k*	7.2	per m	light attenuation coefficient	estimated from field data
*m*	0.0052	per day	relative mortality rate	estimated from field data
*M*_c_	0.1	m	critical mat thickness for floating	estimated from field data
*r*	0.02	per day	relative growth rate	derived from Clymo (1970)

Notes

We chose default parameter settings to mimic average conditions in raised bogs in the Netherlands, based on literature and our field data.

Here, *S* describes the change in thickness of the living peat moss layer, *r* is the relative growth rate per unit of time, and fl is the P‐I (photosynthetic irradiation) curve of peat moss (see Eq. 2). Parameter *K* is carrying capacity for the living peat moss layer, and *m* is its relative mortality rate (see Table 1 for units and default values). The P‐I curve of peat moss is described as

(2) $$\text{fl}=1‐e^{\left(‐I/I_{k}\right)}$$

where *I* is the light availability, and *I_k_* the saturation irradiance constant in PAR. Light availability (*I*) is calculated using the Lambert‐Beer equation

(3) $$I=I_{\left(0\right)}e^{\left(‐k\cdot D\right)}$$

with *k* as the attenuation coefficient and *D* as the depth at which the acrotelm (living top layer) occurs. The change in peat matt thickness (*M*) is described by the second differential equation

(4) $$\frac{\mathrm{dM}}{\mathrm{dt}}=m\times S‐d\times M$$

where *m* is the relative mortality rate of the living peat moss layer *S*, and *d* is the decay rate of the peat mat. Finally, we assume that a peat mat becomes floating once a critical thickness of *M* is reached, causing the depth of the live peat layer to change from *D*_max_ to 0; i.e.

(5) $$D=D_{\max}\;\text{if}\;M<M_{c}\;\text{and}\;D=0\;\;\text{if}\;M\geq M_{c}$$

where *M*_c_ is the critical mat thickness required for floating.

To investigate whether alternative stable states occur in our minimal bog lake simulation model, we explored its sensitivity to the depth of the lake through a bifurcation analysis using GRIND for Matlab. In such an analysis, potential critical transition thresholds and hysteresis (i.e., parameter range where two states can occur) are determined by a numerical procedure in which a key parameter value is increased and subsequently decreased again in small steps (van Nes 2017). In our case, we decreased *D*_max_ from 1 to 0 m in stepwise increments of 0.01 m. At each step, the model was left to stabilize for 10,000 yr. Next, values of *S* and *M* were recorded, after which the value of *D*_max_ was reduced and the next 10,000 yr of simulation followed. Once a *D*_max_ of 0 was reached, we performed the same numerical procedure in the opposite direction.

### Field experiment

**Fig. 3:**
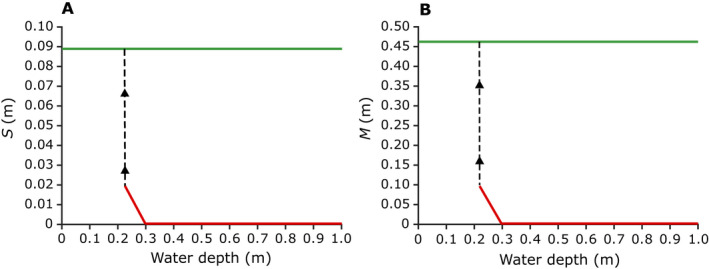
Results of the bifurcation analysis. The occurrence of alternative stable states highlighted by (A) the living peat moss layer (*S*) and (B) the thickness of the peat mat (*M*) in relation to water depths. The solid green lines represent a floating vegetated/peat mat state and the red a bare state (both equilibria are stable beyond a depth of 0.22 m). The dashed black line with the arrows indicates the direction of change, i.e., when water depth is decreased in our model, spontaneous recovery from the bare (red) state towards the vegetated state occurs at 0.22 m.

As hypothesized, our model results suggest that artificial bog lakes can in theory be characterized by two alternative stable states (Fig. 3): open water or a floating peat mat. To further explore this in the field, we used floating biodegradable structures to investigate (1) whether a state shift from open water to floating peat mats can be accomplished as a general test for the occurrence of alternative stable states and (2) if this approach can be applied as a potential restoration measure to overcome critical bottlenecks for peat moss growth. The field experiment was conducted in the Fochteloërveen, a rewetted bog remnant in the Netherlands (Fig. 2, 53°0'22.78" N, 6°22'24.75" E). The bog has been heavily exploited for peat extraction, drainage‐based agriculture, and buckwheat fire culture during the last centuries. Rewetting via dams resulted in a raised water table, and in a higher cover of peat mosses (Altenburg et al. 2017), but also in the formation of large unvegetated lakes. We selected a wave‐sheltered part of a 3‐ha bog lake (the dominant wind direction originates from the southwest in the Netherlands). The water layer in this area was 0.6 ± 0.09 m deep (mean ± SE) at setup (see Appendix S1: Fig. S2 for the water layer depth through time), was highly colored (E_450_ = 0.17), and was low in dissolved CO_2_ concentrations in the water layer (120 µmol/L). The bottom consisted of a 0.69 ± 0.04 m thick peat layer, without any peat mosses, presumably due to light limitation (see Appendix S1: Table S1 for characteristics of the water layer).

#### Experimental setup

The experiment consisted of four treatments, replicated eight times (randomized block design). The treatments consisted of (1) an unmanipulated control, (2) control with peat moss addition, (3) structure with peat moss addition, and (4) structure with organic material and peat moss (Fig. 2F, Appendix S1: Fig. S3 for the layout of the eight replicate blocks in the lake). Plots were constructed in March 2017 and were harvested in July 2019, after a 28‐month experimental period.

Each structure consisted of three stacked biodegradable BESE‐sheets (sheet dimensions: 91.5 × 45.5 × 2 cm, length × width × height; BESE Ecosystem Restoration Products, Culemborg, The Netherlands; Temmink et al. 2020). To create organic matter (OM) modules, we added 2 kg of fresh peat (0.9 kg/L, origin Baltic States) on top of the lowest sheet, after which the second sheet was clicked on top. Next, we added 340 g fresh mass (~10 g dry mass [DM]) *S. cuspidatum* or *S. palustre*, collected in the Fochteloërveen, on top of each two‐sheet OM module (Fig. 2D). Finally, we clicked a third sheet on top, resulting in a 6‐cm thick, three‐layer module. To create modules without OM, we repeated the steps described above, but without the addition of OM. Finally, we randomly combined two modules containing different species into a two‐module plot (Fig. 2E). A two‐module plot always consisted of two species with either a + or − OM treatment. We then attached a PVC tube (2 cm in diameter) around the combined two‐module plot (91.5 × 91 × 6 cm, length × width × height) to ensure floatation (Fig. 2F). We used non‐degradable PVC‐tubes for experimental purposes. We placed the plots 2.5 m apart and secured them between four iron pins, after which we installed caution tape around each plot to minimize bird disturbance (Gahlert et al. 2010). Plots without structures were marked with four iron pins and caution tape was installed. For the “peat moss control treatment,” the same amount of material was introduced into each plot, while no peat mosses were introduced in the “controls.” In the “peat moss control,” the added peat mosses immediately floated away and washed ashore on the same day. Therefore, this treatment was omitted from further analyses.

#### Sample analyses

At every field visit (*n* = 16, frequency once every one to two months), surface water level was determined using a fixed beacon. In addition, we took surface water samples with a 1‐L cup attached to a 2‐m long pole to prevent disturbances. The conductivity of the surface water was measured in situ (TetraCon 925 probe connected to a Multi 3,420 m; WTW, Weilheim, Germany). In the laboratory, the pH and alkalinity were measured with an Ag/AgCl electrode (Orion, Thermo Fisher Scientific, Waltham, Massachusetts, USA) coupled to an 877 Titrino plus (Metrohm, Herisau, Switzerland). Total Inorganic Carbon (TIC) was measured using an infrared carbon analyzer (IRGA; ABB Analytical, Frankfurt, Germany), after which bicarbonate (${\text{HCO}}_{3}^{‐}$) and CO_2_ were calculated based on the pH equilibrium (e.g., van Bergen et al. 2020). A 10‐mL subsample of each filtered water sample (Glass microfiber filters, outer diameter 47 mm, GF/C, Whatman, GE Healthcare UK Limited, Little Chalfont, Buckinghamshire, UK) was conserved by adding 0.1 mL of nitric acid (${\text{HNO}}_{3}^{‐}$ 65%) and stored at 4°C until P analysis by inductively coupled plasma emission spectrophotometry (ICP‐OES; model IRIS Intrepid II XDL, Thermo Fisher Scientific, Franklin, Tennessee, USA). The rest of each sample was stored in polyethylene bottles at −20°C prior to analyses. Nitrate (${\text{NO}}_{3}^{‐}$), ammonium (${\text{NH}}_{4}^{+}$), and phosphate (${\text{PO}}_{4}^{3‐}$) were measured colorimetrically with an auto analyzer (Auto Analyzer III, Bran and Luebbe GmbH, Norderstedt, Germany). Potassium (K) was determined by flame photometry (FLM3 Flame Photometer, Radiometer, Copenhagen, Denmark). Extinction at 450 nm was measured (Double beam, UV‐Vis‐spectrophotometer, UV‐6300PC, VWR, Amsterdam, the Netherlands) as an estimate of humic substance concentration (Kirk 1994, Smolders et al. 2003).

After a period of 28 months, we took a photograph of each plot to determine the lateral expansion of the vegetation using ImageJ. Furthermore, we measured the peat moss lawn height relative to the water table in each plot, after which we transported the intact plots to the lab in July 2019. We harvested the homogeneous occurring *S. cuspidatum* from the inundated peat in controls and calculated the biomass for the subplots, because they were situated on the sediment and not visible due to the low water clarity. Next, we collected and sorted peat mosses and vascular plants from each module of each two‐module plot, determined fresh mass and dry mass (70°C until constant mass).

#### Statistical analyses

Since only one species survived the treatments (*S. cuspidatum*) and no *S. palustre* was found after 28 months (Appendix S1: Fig. S3), the effect of peat moss species was not taken into account when analyzing the data. To test whether establishment structures with or without organic matter stimulate peat moss peat moss biomass, height, and vascular plant biomass (*Agrostis canina*), we analyzed the data with general linear mixed models with a Gaussian distribution and block as random effect. These analyses were followed by Tukey post‐hoc test (Lenth and Lenth 2018). Data were square root, reciprocal and log transformed for peat moss biomass, peat moss height and *Agrostis canina* biomass, respectively. To test the effect of treatment on *Juncus effusus* biomass, we used a zero‐inflated Gaussian mixed model, as the data had many zeros (package NBZIMM). To test whether lateral expansion differed between structures with or without OM, we analyzed the data with a Student’s *t* test. Surface water quality data were averaged, and the minimum and maximum values were determined (Appendix S1: Table S1). All analyses were performed in R (version 3.6) statistical and programming environment (R Core Team 2020). All results are shown with their standard error of the arithmetic mean (±SE) and the significance level is at *P* < 0.05.

## Results

### Model results

The model shows that spontaneous peat moss growth is only possible when the water depth is between 0 and 0.29 m (Fig. 3). Between 0.22 and 0.29 m depth the model shows bistability between a 9‐cm thick living peat layer (Fig. 3A) on top of a 0.46‐cm floating peat mat (Fig. 3B) vs. a thin (maximum 0.02 cm) layer of living peat moss with a thin (maximum 0.1 cm) peat mat that is insufficient to float. Beyond a depth of 0.29 m, peat moss is not able to grow at the bottom of the lake at all. Here, peat moss is only able to grow when already afloat, yielding alternative stable states between a bare state and a state with a floating mat that is able to sustain itself irrespective of the water depth.

### Field results

**Fig. 4:**
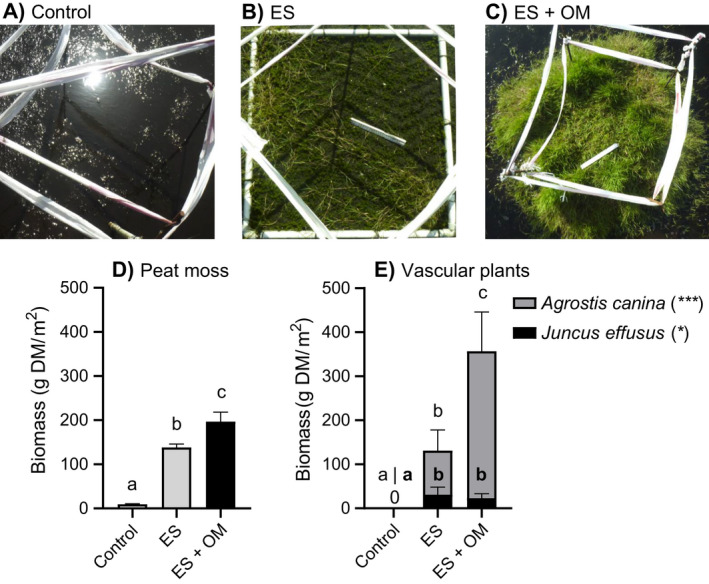
Peat moss and vascular plant biomass. Pictures of the three treatments after 28 months (A, control; B, structure; C, structure with organic material). (D) Peat moss *Sphagnum* *cuspidatum* and (E) vascular plant biomass (*n* = 16; DM, dry mass) in controls, structures (ES) and structures with organic material (OM). Error bars represent SE. Significant contrasts are indicated by different letters. Asterisks indicate significant treatment effect on vascular plant biomass for *Agrostis canina* (***P < 0.001) and *Juncus effusus* (*P = 0.01) with different letters for significant contrasts for each species.

Structures positively affected peat moss and vascular plant biomass in the unvegetated human‐made lake (Fig. 4), and this effect was enhanced by the addition of organic material (OM). The surface water was characterized by low concentrations of CO_2_ (120 ± 10 µmol/L, mean ± SE), ${\text{HCO}}_{3}^{‐}$ (0.9 ± 0.09 µmol/L), ${\text{NH}}_{4}^{+}$ (32 ± 6 µmol/L) and was highly colored (0.17 ± 0 E_450_, Appendix S1: Table S1 for other parameters). In general, peat moss biomass was lowest in controls with 9.4 ± 1 g DM/m^2^ (*F*_2,38_ = 153.8, *P* < 0.001, Fig. 4A), intermediate in structures without OM (138 ± 8 g DM/m^2^) and highest in structures with OM (197 ± 21 g DM/m^2^). Similar to the peat moss results, vascular plant biomass, consisting almost exclusively of *Agrostis canina*, was highest in structures with OM (334 ± 90 g DM/m^2^, Fig. 4B), 3.3 times lower in structures without OM (100 ± 50 g DM/m^2^), and absent in controls (0 ± 0 g DM/m^2^, *F*_2,38_ = 40.6, *P* < 0.001). *Juncus effusus* biomass was absent in the controls (0 ± 0) and was highest in the structures with (31 ± 17 g DM/m^2^) and without OM (23 ± 10 g DM/m^2^, *F*_2,38_ = 4.8, *P* = 0.0136).

**Fig. 5:**
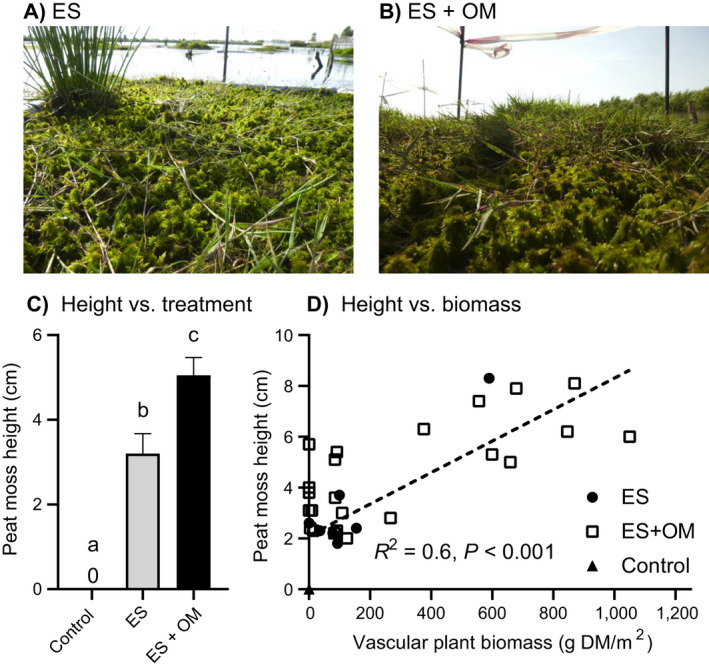
Peat moss height in treatments and related to vascular plant biomass. Pictures of the difference in peat moss (*Sphagnum* *cuspidatum*) height in the structures (A) without and (B) with organic material. (C) Peat moss height relative to the water level (cm) in controls, structures (ES), and structures with organic material (OM), and the (D) relationship between peat moss height (cm) and vascular plant biomass. Error bars represent SE. Significant contrasts are indicated by different letters. The regression line was fitted using all data.

Peat moss (*S. cuspidatum*) height was positively affected by structures, and particularly by structures with OM, while peat mosses in controls did not grow vertically (Fig. 5, *F*_2,38_ = 1272.4, *P* < 0.001). In structures without OM, peat mosses reached a height of 3.2 ± 0.5 cm, while in those with OM the moss layer was 1.6 times higher (5.1 ± 0.4 cm;). Interestingly, peat moss height was positively related to the vascular plant biomass (*R* ^2^ = 0.6, *P* < 0.001). *A. canina* accompanied by *S. cuspidatum* laterally expanded 53 ± 6 cm outside of the structure, irrespective of OM treatment (*P* = 0.36).

## Discussion

**Fig. 6:**
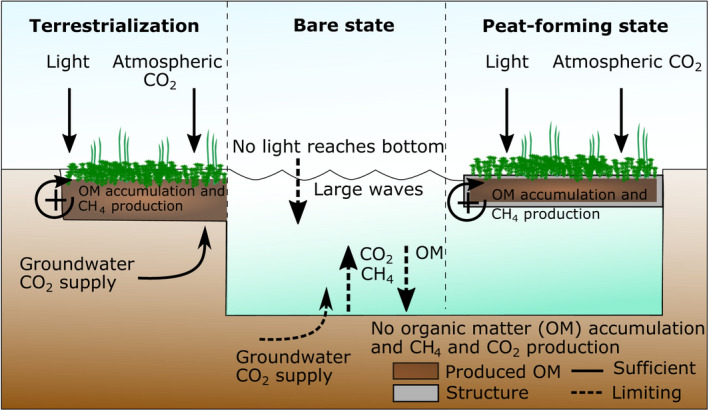
Alternative stable states in artificial bog lakes. Natural terrestrialization (left), bare state (middle) and peat‐forming vegetated state on floating mat mimics (right). Vegetation is able to develop as it has ample light and carbon for primary growth and is not physically stressed by wave action (left), while these factor limit growth in a deep lake (middle).

Human‐made bog lakes in many restored bog remnants in Europe and North America, originate from former peat extraction and subsequent rewetting. These waters are often largely without vegetation, most likely due to a combination of unfavorable abiotic conditions and establishment thresholds caused by a lack of positive feedbacks normally generated by the vegetation itself (Paffen and Roelofs 1991, Smolders et al. 2002, 2003, Tomassen et al. 2003*b*). Using a minimal model, we show how these bog lakes can display alternative stable state behavior between contrasting bare open water and vegetated floating mat states driven by light availability, which is in agreement with our hypothesis. Given this finding, we conducted a field experiment to investigate whether such states exist in the field, and simultaneously test a novel method to induce a state shift from open water to peat‐forming vegetation as a restoration measure. In accordance with our hypotheses, the floating biodegradable structures aid in overcoming light and CO_2_ limitation and wave stress bottlenecks, allowing the re‐establishment of self‐facilitating mechanisms (Fig. 6). Moreover, organic material further stimulated vascular plant and peat moss growth. In establishing floating peat mosses, we also provide proof of concept for our approach as a restoration method, which has as advantages that it does not require large‐scale white peat transplantation.

### Alternative stable states in bogs

In bog remnants, such as our study site, the formation of deep lakes after rewetting is unnatural. However, bog lakes can also be a natural phenomenon, particularly in regions with an excess of moisture (Weber 1902, Foster et al. 1988). They can deepen because the rate of peat accumulation in the surrounding mire exceeds the rate of sedimentation/organic matter accumulation in the lake, and it can expand due to wave action (Clymo 1970, Foster et al. 1988, Crushell et al. 2009). In our model, natural colonization of open water by peat mosses occurs at water depths <0.29 m. This is in agreement with findings from the field of Smolders et al. (2003) and Money (1995). They indeed indicated that at shallow water depth (<0.3 m), particularly when the water is highly colored, submerged growing peat mosses have sufficient access to light to grow and form mats. However, this depth threshold for natural recovery is site specific as local factors, including suspended particles and dissolved organic compounds, control light extinction and thus light availability at a certain depth (Smolders et al. 2003). For instance, the 5% light threshold required for plant growth may reach a bog‐pool bottom of 5.4 m in Norway (E_450_ of <0.05), 0.85 m in Clara bog Ireland (E_450_ of 0.06), while this threshold occurs at only 0.2 m at the Bargerveen in the Netherlands (E_450_ of 0.26) (Limpens et al. 2019). As comparison, at our site, the E_450_ ranges between 0.1 and 0.27 (0.17 on average, Appendix S1: Table S1).

Once light is sufficient, the concentration of dissolved CO_2_ in the water column can become the next limiting factor controlling submerged peat moss growth (Paffen and Roelofs 1991, Smolders et al. 2003, Patberg et al. 2013). Moreover, once mats are afloat, they also need to be sufficiently coherent to resist wind‐driven waves. For simplicity, however, these additional factors were not addressed in our minimal model. Yet, even without these potentially aggravating factors our model clearly demonstrates the potential for occurrence of alternative stable states as a function of water depth and light availability. This, to our knowledge, has not yet been described in literature for such systems. The occurrence of two stable states typically results in systems that are challenging to restore, because of strong feedbacks (Scheffer et al. 2001, van der Heide et al. 2010). Therefore, restoration practitioners can utilize our work to either fine‐tune local hydrology to promote natural peat moss growth and peat formation, or apply alternative approaches to initiate a state shift from open water to a floating peat mat.

### Initiating a state shift using establishment structures

We found that the floating structures successfully ameliorated light and CO_2_ limitation, and wind‐driven waves, factors that suppresses the peat growth mosses and may therefore induce alternative stable state dynamics (Paffen and Roelofs 1991). At our site, water color measurements indicate that 5% of the light can only reach the bottom when water depth is below 0.29 m (Smolders et al. 2003). In addition, CO_2_ concentrations (120 ± 10 µmol/L) lay substantially below the threshold (>750 µmol/L) required to form a floating mat (Paffen and Roelofs 1991). High levels of ${\text{NO}}_{3}^{‐}$, ${\text{NH}}_{4}^{+}$, and ${\text{HCO}}_{3}^{‐}$ (>250–500 µmol/L) in the surface water have also been found to negatively affect peat moss growth (Press et al. 1986, Harpenslager et al. 2015, Koks et al. 2019). However, in our lake, ${\text{NO}}_{3}^{‐}$ and ${\text{HCO}}_{3}^{‐}$ (both <5 µmol/L) were virtually absent, while water dissolved ${\text{NH}}_{4}^{+}$ concentrations (~32 µmol/L) remained below levels that impair growth (>200 µmol/L, Appendix S1: Table S1; Press et al. 1986, Rudolph and Voigt 1986). While not the case at our site, guanotrophy from gulls and other water birds is known to affect vegetation composition in large bodies of open water in peat cuttings (Tomassen et al. 2005). This may substantially lower water quality and may lead to the accumulation of N and P and algal growth (Leentvaar 1967).

Interestingly, organic matter added to structures facilitated vascular plant biomass, and they in turn facilitated peat moss biomass and vertical growth by providing physical support for growth. Vascular plants are known to facilitate peat moss length increment, as they provide structure and shade (Malmer et al. 1994, Pouliot et al. 2011). The high vascular plants biomass in this treatment may be explained by a higher supply of nutrients as well as the availability of rooting substrate by the organic material, combined with the high atmospheric nitrogen deposition in the Netherlands (Velders et al. 2018). In addition, *A. canina* started growing outside the structure and provided structure to *S. cuspidatum* (Fig. 4C). This may indicate that *A. canina* enables the expansion of peat mosses outside the structure and pushing the system into a vegetated state. Next to providing structure for peat mosses, these larger vegetation patches can also break up waves that enables the attached mosses to resist wave energy. Otherwise, wind‐induced waves in deep and large bog lakes might break up the newly established mosses. This means that the artificial structures can act as nucleus for the growth of more extensive mats, without covering the entire lake with structures, which may substantially lower restoration costs. Based on this observation and materials used for the current small‐scale experiment, we created four scenarios differing in initial coverage by the structure and estimate that the cost to cover 10%, 25%, 50%, or 100% of a lake are 34.4, 86, 172, and 344 thousand $US/ha, respectively (for details see Appendix S1: Table S2). Moreover, the peat moss to vascular plant ratio shifts from 1.05 in structures to 0.55 in structures with organic material. Although vascular plants facilitated peat moss height in the short term, this may well lead to competition and light limitation for the peat mosses in the long term (Hayward and Clymo 1983, Hogg et al. 1995, Tomassen et al. 2003*a*). When peat mosses are outcompeted by vascular plants, this might lower the potential of the new system to store carbon, as vascular plants degrade more readily than peat mosses (Mettrop et al. 2014).

Our study shows that proper species selection in relation to the targeted environment is of utmost importance for success as peat moss biomass consisted only of *S. cuspidatum* at the end of the experimental period. The habitat created, 3 cm below the water level, is more suitable to *S. cuspidatum* compared to *S. palustre,* as the latter species typically grows in drier conditions (Daniels and Eddy 1985). The favorable conditions for *S. cuspidatum* growth, allowed this species to colonize the substrate inoculated by *S. palustre* (Appendix S1: Fig. S4). *S. palustre* or other late‐succession species may benefit from drier conditions at onset (i.e., a shallower support structure), or from a two‐step restoration approach (Money 1995). In the latter approach, faster growing pioneer species are first facilitated by the structures enabling the fast formation of a peat lawn and a floating mat. After successful establishment and floating mat formation, late‐successional species can then be introduced as a second step, demonstrated by Smolders et al. (2003) for *S. magellanicum* on a well‐developed carpet of *S. cuspidatum*. In this way, late‐successional species profit from drier conditions, allowing them to gradually create a typical raised bog vegetation community (Robroek et al. 2009). The formation of such a mat may occur rather rapidly. For instance, *Sphagnum* is able to form a dense layer consisting of living and dead material of 18 cm thick in under 10 yr (Vroom et al. 2020).

### Conclusion and implications for restoration

In this paper, we demonstrate the potential for alternative stable states, open water vs. floating peat mat, in artificial lakes in bog remnants, using a minimal model, and confirm this in our field experiment. Moreover, we show that restoration by inducing a state shift from open water to floating mats using biodegradable establishment structures can be achieved without the need to introduce unsustainable peat. By introducing peat mosses close to the water surface in the structures light and carbon limitation, and wave stress were ameliorated, thereby overcoming establishment thresholds. The shift from open water to peat‐forming vegetation may eventually result in carbon storage, as bog vegetation sequesters and stores more carbon than open water (Couwenberg et al. 2011). Such as shift may aid in climate mitigation when it takes places on a sufficiently large scale in many bog remnants worldwide. Moreover, the model results can also be used to fine‐tune local hydrology to optimize peat moss growth. We further observed that the structures act as a nucleus from where terrestrialization can occur (Fig. 4C). It is important to note, that for widespread upscaling a sustainable donor supply could be an issue, because peat mosses are relatively scarce in the Netherlands and Western Europe. Mosses grown at *Sphagnum* farms can be a sustainable alternative to mosses collected in natural peatlands (Gaudig et al. 2017, Temmink et al. 2017, Vroom et al. 2020). In addition, natural succession of a floating peat moss carpet towards an ombrotrophic bog vegetation can be slow process (decades to a century; Lindsay and Clough 2016), and the introduction of later‐successional peat mosses on top of floating mats may speed this up. Lastly, the results we show here may well be extended to the restoration of other peat ecosystems that are characterized by a lack of vegetation development due to unfavorable environmental conditions. Clear examples are fens where the colonization of open water by terrestrial species is crucial to form floating mats (Sarneel 2010) and facilitate brown moss (*Scorpidium* sp.) growth establishment (Lamers et al. 2002, Lamers et al. 2015). Restoration techniques such as the one presented here may become important to overcome establishment thresholds and achieve greater restoration success in degraded peatlands.

## Supporting information

Appendix S1

## Data Availability

Data (Temmink et al. 2021) that support the main findings of this study are available via Data Archiving and Networked Services (DANS) EASY at: https://doi.org/10.17026/dans‐xg4‐9mz7.
