# Supplementary material for: Overcoming establishment thresholds for peat mosses in human‐made bog pools
Source: Ecol Appl. 2021 May 30;31(6):e02359. doi: 10.1002/eap.2359 (PMC8459249; doi:10.1002/eap.2359)
Supplement: Supplementary file 1 — Appendix S1 [file EAP-31-e02359-s001.pdf]

Supporting Information. Temmink, R.J.M., P.M.J.M. Cruijsen, A.J.P. Smolders, T.J. Bouma, G.S. Fivash, W. Lengkeek, K. Didderen, L.P.M. Lamers, and T. van der Heide. 2021. Overcoming establishment thresholds for peat mosses in human-made bog pools. Ecological Applications.

Appendix S1

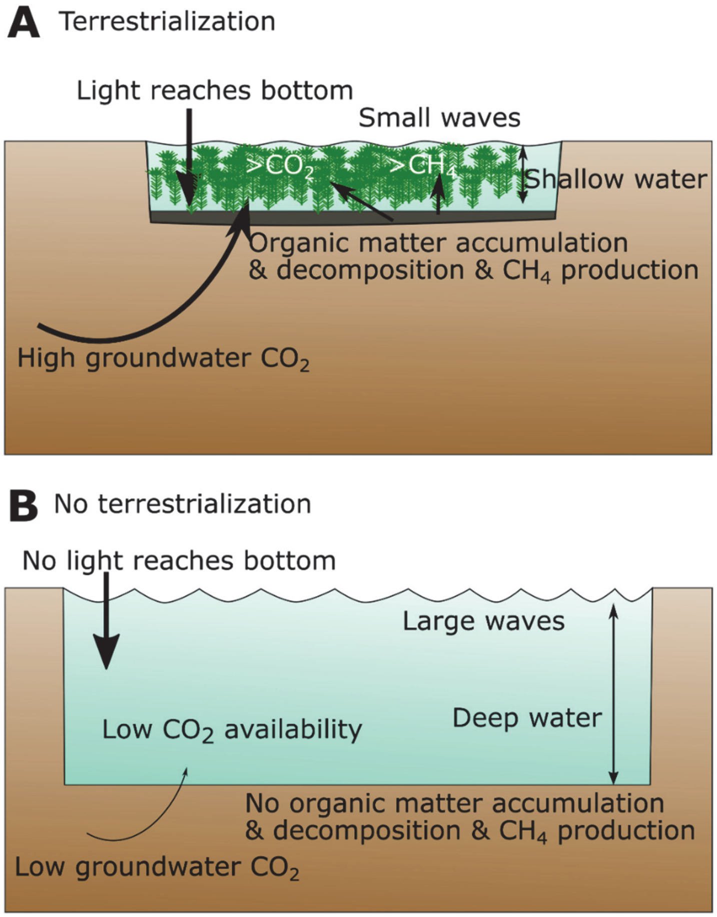

Fig. S1. Terrestrialization of a bog lake. Schematic view of different "starting" conditions with (A) successful and (B) unsuccessful terrestrialization of deeply inundated lagoons. Typically, terrestrialization starts when sufficient light (shallow water) and CO_2 is available for peatmoss growth originating either from carbon-rich groundwater or from organic matter decomposition. Stimulated peatmoss growth inducing internal positive feedbacks concerning CO_2 (organic matter decomposition) and indirectly light availability (organic matter accumulation). Terrestrialization does not start when waters are too deep (light limited), CO_2 limited or are too large (wind-driven waves).

Table S1. Water quantity and quality. Water quantity and quality of the experimental pool in Fochtelöerveen during the experimental period (March 2017 until July 2019).

**Parameter**	**Unit**	**Average ± SEs (*n*)**	**Min**	**Max**
Water depth	cm	39 ± 3 (19)	16.5	58
Electrical conductivity	µS cm^{-1}	114 (33)	84	137
Water color	E_{450}	0.17 ± 0 (96)	0.1	0.27
pH		4.2 ± 0.02 (96)	3.6	4.7
Alkalinity	meq L^{-1}	0.01 ± 0 (96)	0	0.1
CO_2	µmol L^{-1}	120 ± 10 (96)	23	550
HCO_3^-	µmol L^{-1}	0.9 ± 0.09 (96)	0	5
NH_4^+	µmol L^{-1}	32 ± 6 (100)	0.3	280
NO_3^-	µmol L^{-1}	4 ± 0.3 (100)	0	11
PO_4^{3-}	µmol L^{-1}	2 ± 0.2 (100)	0	10
P	µmol L^{-1}	7 ± 2 (99)	0.2	71
K	µmol L^{-1}	36 ± 3 (99)	10	100

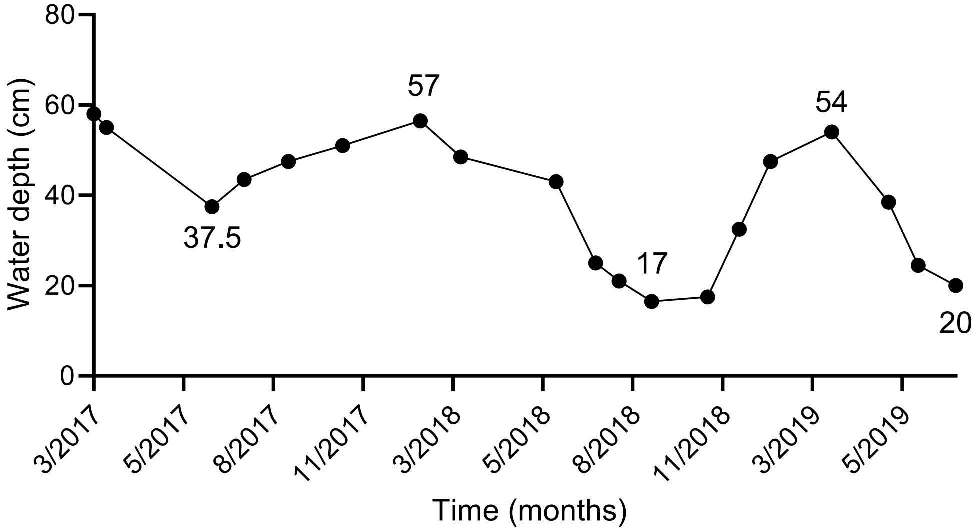

Fig. S2. Water depth fluctuation during the experimental period. During the first summer the water level dropped with from 59 cm to 37.5 cm in 2017, but during two severe summer drought episodes, the water level reached 17 and 20 cm in 2018 and 2019, respectively.

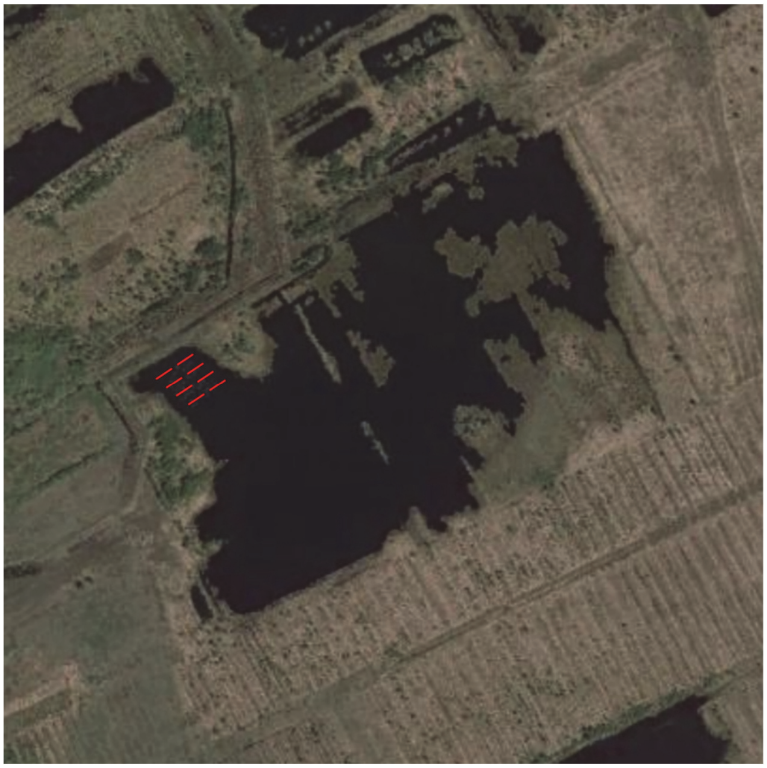

Figure S3. Layout of the eight replicate blocks in the bog lake.

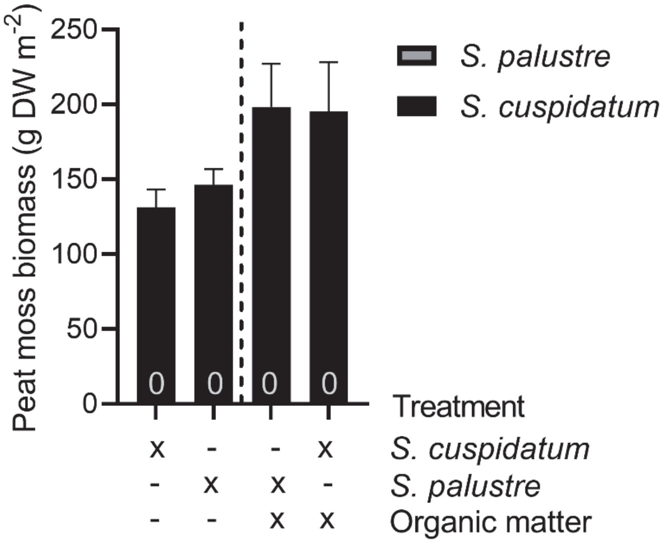

Fig. S4. Effect of treatments on peat moss species. Peat moss (*S. cuspidatum*) biomass (**A**, g DW m^{-2}, n = 8, error bars represents SEs) of *S. cuspidatum* and *S. palustre* grown in establishment structures (ES) with and without organic material (OM). The zero's represent the biomass of *S. palustre*.

Table S2. Cost-estimate for the used method. To illustrate the potential applicability of our approach, we calculated construction costs for a number of scenarios in which we upscale our specific technique as an example. Specifically, we considered the following four scenarios: 10, 25, 50 and 100% initial cover. We chose these scenarios, because our data revealed that the structures can act as nuclei for growth (vegetation expanded 53 ± 6 cm out of the structures during the experimental period). Depending on the timeframe for restoration, different initial coverages can be used. Construction costs are extrapolated from actual costs in our experiments. In each scenario, the approximately 1-m^2 structures were assumed to be spread out evenly across space.

**Scenario 1**: Low initial cover			**Scenario 2**: Low-medium initial cover		
Initial cover (%) | Plots (# ha^{-1})	Costs (US k$)	Category	Initial cover (%) | Plots (# ha^{-1})	Costs (US k$)	Category
10 % | 1000	24	BESE[a]	25% | 2500	60	BESE[a]
	3	PVC-tube[b]		7.5	PVC-tube[b]
	0.4	Moss[c]		1	Moss[c]
	7	Labor[d]		17.5	Labor[d]
	**34.4**	**Total**		**86**	**Total**
**Scenario 3**: Medium initial cover			**Scenario 4**: Completely covered		
Initial cover (%) | Plots (# ha^{-1})	Costs (US k$)	Category	Initial cover (%) | Plots (# ha^{-1})	Costs (US k$)	Category
50 % | 5000	120	BESE[a]	100 % | 10000	240	BESE[a]
	15	PVC-tube[b]		30	PVC-tube[b]
	2	Moss[c]		4	Moss[c]
	35	Labor[d]		70	Labor[d]
	**172**	**Total**		**344**	**Total**

[a]module is 3 sheets thick, 2 modules wide (dimension sheet 92x45.5x2.0 cm), 4 US $ per sheet.
[b]4 meter of PVC-tube per structure, 1.3 US $ per m (for providing buoyancy).
[c]20 g DW peatmoss m^{-2} at a price of 20 $ US kg^{-1} (Wichmann et al. 2020).
[d]Construction time plus organization/project planning time (0.1 + 0.1) an hourly rate of 35 US$.

Literature Cited

Wichmann, S., M. Krebs, S. Kumar, and G. Gaudig. 2020. Paludiculture on former bog grassland: Profitability of Sphagnum farming in North West Germany. Mires and Peat, 26(08).
